# Stretching positions for the coracohumeral ligament: Strain measurement during passive motion using fresh/frozen cadaver shoulders

**DOI:** 10.1186/1758-2555-3-2

**Published:** 2011-01-19

**Authors:** Tomoki Izumi, Mitsuhiro Aoki, Yoshitaka Tanaka, Eiichi Uchiyama, Daisuke Suzuki, Shigenori Miyamoto, Mineko Fujimiya

**Affiliations:** 1 Doctoral Course of Physical Therapy, Graduate School of Health Sciences, Sapporo Medical University, Sapporo, Japan; 2 Department of Orthopaedic Surgery, Sapporo Daiichi Hospital, Sapporo, Japan; 3 Department of Orthopaedic Surgery, Japan Self Defence Force Sapporo General Hospital, Sapporo, Japan; 4 Department of Physical Therapy, Sapporo Medical University, Sapporo, Japan; 5 2nd Department of Anatomy, Sapporo Medical University, Sapporo, Japan; 6 Department of Physical Therapy, Hokkaido Bunkyo University, Eniwa, Japan

## Abstract

**Background:**

Contracture of the coracohumeral ligament is reported to restrict external rotation of the shoulder with arm at the side and restrict posterior-inferior shift of the humeral head. The contracture is supposed to restrict range of motion of the glenohumeral joint.

**Methods:**

To obtain stretching position of the coracohumeral ligament, strain on the ligament was measured at the superficial fibers of the ligament using 9 fresh/frozen cadaver shoulders. By sequential measurement using a strain gauge, the ligament strain was measured from reference length (L0). Shoulder positions were determined using a 3 Space Tracker System. Through a combination of previously reported coracohumeral stretching positions and those observed in preliminary measurement, ligament strain were measured by passive external rotation from 10° internal rotation, by adding each 10° external rotation, to maximal external rotation.

**Results:**

Stretching positions in which significantly larger strain were obtained compared to the L0 values were 0° elevation in scapula plane with 40°, 50° and maximum external rotation (5.68%, 7.2%, 7.87%), 30° extension with 50°, maximum external rotation (4.20%, 4.79%), and 30° extension + adduction with 30°, 40°, 50° and maximum external rotation (4.09%, 4.67%, 4.78%, 5.05%)(P < 0.05). No positive strain on the coracohumeral ligament was observed for the previously reported stretching positions; ie, 90° abduction with external rotation or flexion with external rotation.

**Conclusions:**

Significant strain of the coracohumeral ligament will be achieved by passive external rotation at lower shoulder elevations, extension, and extension with adduction.

## Background

External rotation of the glenohumeral joint is important to maintain activity of daily living and it contributes to allow flexion and abduction of the shoulder joint.[1] Patients suffering a frozen shoulder or rotator cuff tear demonstrate restriction of abduction and external rotation of the glenohumeral joint.[1,2] For such cases, physiotherapy may help to stretch the soft tissue and maintain mobility of the glenohumeral joint.[3]

Harryman et al[4] reported that external rotation of the glenohumeral joint at 0 degrees of elevation was reduced by approximately 50% in patients with contracture of the rotator interval involving the coracohumeral ligament. Previous authors have also reported that contracture of the rotator interval reduced external rotation at 0 degrees of elevation,[2,5] and external rotation in flexion and abduction.[4,6,7] Improvement of abduction and flexion of the glenohumeral joint was obtained by surgical release of the coracohumeral ligament in patients who had severe contracture of the joint.[5,7] Neer et al[5] and Gagey et al[8] reported that the coracohumeral ligament played a role in limiting the range of motion for external rotation of the glenohumeral joint. Bowen et al[9] and Warner et al[10], however, reported that the ligament acted to limit the inferior shift of the humeral head, whereas, Harryman et al[4] reported that the ligament limited the posterior shift of the humeral head.

In physiotherapy, it has been experienced that elongation of the coracohumeral ligament in the rotator interval can provide an increase in the range of external rotation and flexion of the glenohumeral joint. Thus, we consider that non-invasive stretching of the coracohumeral ligament may not only improve the range of motion of the glenohumeral joint but reduce pain due to contracture of the joint.

Various stretching procedures for the coracohumeral ligament have been reported in literature; however, no consensus has been reached [11-16]. These stretching procedures have been obtained from observations of the anatomical location and length of the ligament and capsule, and have been used as a standard in physiotherapy. However, they were not verified by the measurement of the strain. Therefore, it is important to measure the strain on the coracohumeral ligament to disclose appropriate stretching positions for the coracohumeral ligament.[11,13-17]

In this study, nine fresh/frozen cadaver shoulders were used to perform strain measurement and determine stretch positions for the coracohumeral ligament. Strain on the coracohumeral ligament was measured during passive external rotation at nine designated shoulder positions, in which the reference length (L0) of the ligaments was taken into consideration. The purpose of this study is to indicate procedures for potential stretch positions for the coracohumeral ligament in vivo.

## Materials and methods

### Preparation and Specimens

Nine fresh/frozen shoulder specimens (6 males, 3 females) without evidence of tendon rupture or osteoarthritis were used in this experiment. In this study, we observed articular cartilage of the humeral head and glenoid fossa of the scapula after the study, and specimens with osteophytes or abrasion of the articular cartilage were excluded.[18] The age of specimens at death ranged from 79 to 96 years (mean, 86.3 years). Within 24 hours after death, the specimens were transferred from regional hospitals to the 2^nd ^Department of Anatomy of our university. Shoulder specimens were disarticulated from the thorax and kept in a freezer at -20°C. Thawing of the shoulder specimens at room temperature (22°C) was started 12 hours before preparation.

The skin, fascia, muscles, nerves, and vessels were removed sparing the rotator cuff and coracohumeral ligament. In addition, the coracoacromial ligament and anterior part of the acromion were removed, and the coracohumeral ligament and tendon of supraspinatus were exposed. The distal third of the humerus was exposed, and an acrylic stick was inserted perpendicular to the humerus shaft, indicating the direction of the forearm. Next, the humerus was amputated above the elbow. During the experiment, the specimens were kept moist by spraying with saline every 5 to 10 minutes. Room temperature was maintained at 22°C.

### Testing Apparatus

**Figure 1:**
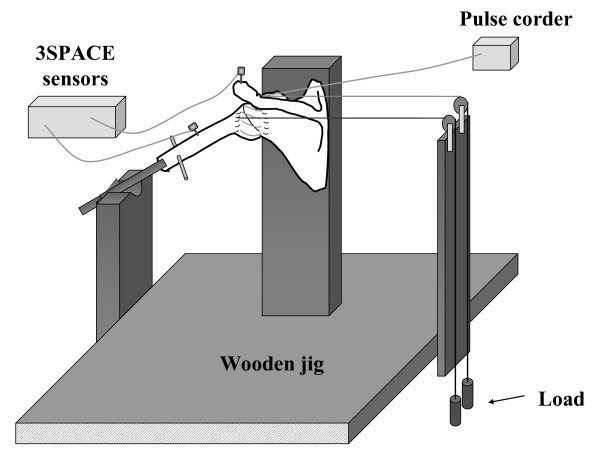
**Experimental set-up**. A displacement sensor was attached to the superficial fibers of the coracohumeral ligament. A six-degree of freedom electromagnetic tracking device was used to monitor the glenohumeral rotational angles.

A wooden jig, consisting of a wooden board and a square post/column (height 500 mm × width 160 mm × thickness 24 mm), was used for this experiment. The ventral surface of the scapula of the specimen was fixed to the wooden post/column so that the medial border of the scapula was perpendicular to the ground to simulate the resting scapular position (Figure 1). Two anchors (Fastin RC threaded suture anchor, Mitek, Massachusetts, USA) were inserted into the bony insertion of the subscapularis tendon and infraspinatus tendon to apply a compression force of 11N (total 22N) via a suture against the glenoid fossa. In previous cadaveric studies, this compression force was used as the minimum force required to prevent subluxation of the humeral head from the glenoid fossa during passive glenohumeral motion.[4,19-22] In this study, humeral head subluxation was carefully observed by three researchers and no visible and palpable subluxation was detected during experiment. A thin wooden rod (400 mm in length) was then inserted into the medullary canal of the humerus to help in maintaining glenohumeral elevation, abduction, flexion, and extension at the designated angle during passive external rotation of the humerus.

### Measurement device

**Figure 2:**
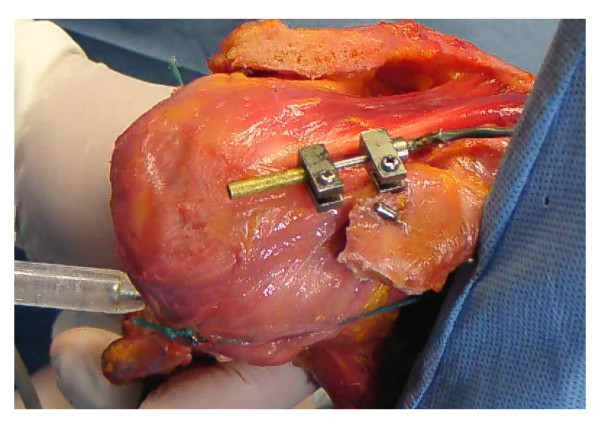
**A photograph of the displacement sensor**. The sensor was attached to the center of the superficial fibers of the coracohumeral ligament parallel to the ligament fibers.

The strain data for the coracohumeral ligament was obtained from a displacement sensor (Pulse Coder, LEVEX, Kyoto, Japan). The linearity error and repetitive accuracy of the Pulse Coder are below 1% and 2.5 μm, respectively. The stroke of the Pulse Coder at this linearity is 14 mm, and all strain measurements were performed within this stroke range. The Pulse Coder consisted of coil sensor and a brass pipe, in which the rod of the coil sensor located. In previous mechanical studies, strain on the ligament and tendon were measured using DVRT (Differential Variable Reluctance Transducer, Microstrain, Williston, Vermont). The mass effect of DVRT was not described in previous reports;[19-21,23,24] however, it can be neglected as the weight of the Pulse Corder is 10.16 g. The sensors were attached to the center of the superficial fiber of the coracohumeral ligament,[11,25,26] and were placed parallel to the ligament fibers (Figure 2).

A six-degree of freedom electromagnetic tracking device (3SPACE FASTRACK, Polhemus, Colchester, Vermont) was used to monitor the glenohumeral angles during measurement.[27] The length, width, height and weight of the Polhemus Sensors were 2.3 cm, 2.8 cm, 1.5 cm and 17 g, respectively.[27] Polhemus sensors were fixed on the bone with titanic screws. This form of fixation is rigid. Stability of the sensor was observed in the monitor. The angle of the glenohumeral joint was simulated by the angle between the plane of the glenoid fossa and the longitudinal line of the humerus. Rotational angle was simulated by rotation of humerus along its longitudinal axis [28]. The glenoid fossa tilts 4 degrees superiorly toward the medial border of the scapula, and at 7 degrees of retroversion [28]. As the scapula of the specimen was fixed on the wooden jig, adjusting the anterior surface of the scapular so as to be parallel to the frontal plane, allows the plane of the glenoid fossa to be determined on the basis of anatomical knowledge. This device enabled measurement of the three-dimensional position and orientation of the sensors relative to the absolute coordinates generated by the source. One sensor was placed on the acromion and the other was placed on the middle portion of the humerus. In this system, angle of the arm flexion, abduction and extension was defined as the angle between the plane of the glenoid fossa and the longitudinal axis of the humerus. The rotation angle was defined as the rotation of the humerus along its longitudinal axis. Within a 750 mm range from the source, the positional accuracy was 0.8 mm root-mean-square, and the angular accuracy was 0.5° root-mean-square.[27]

### Experimental procedure

#### Measurement portion

The coracohumeral ligament is reported to run from the base of the coracoid process to the greater and lesser tuberosities of the humerus.[11] The coracohumeral ligament is classified into superficial and deep fibers, the former inserts into the greater tuberosity and the latter inserts into the lesser tuberosity.[25,26] In this experiment, strain on the superficial fibers, but not the deep fibers, of the coracohumeral ligament was measured, because superficial fibers are considered to from the major part of this ligament.[12] In this study, strain was measured on the central superficial fibers of the coracohumeral ligament as Bigliani et al reported that tensile properties were measured at the center of the inferior glenohumeral ligament,[29] and Noyes et al[30] reported that tensile properties were the highest at the center of the patellar tendon, and those values were used to represent the whole patella tendon. (Figure 2)

#### Measurement positions

**Figure 3:**
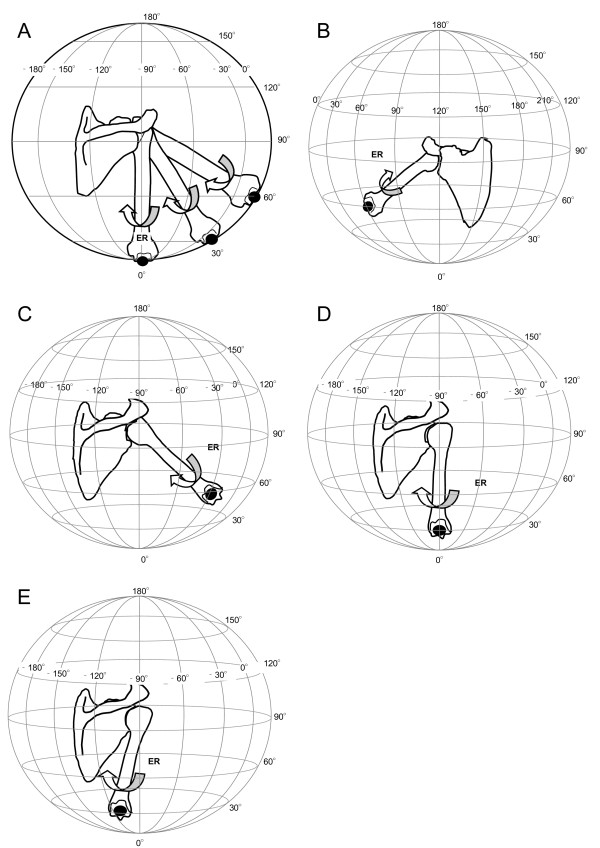
**Measurement positions for the glenohumeral joint in vitro**. External rotation at 0°, 30° and 60° elevation in the scapula plane (Figure 3-A), external rotation at 30° and 60° flexion (Figure 3-B), external rotation at 30° and 60° abduction (Figure 3-C), external rotation at 30° extension (Figure 3-D), external rotation and adduction at 30° extension (Figure 3-E). In the globe system, the scapula plane was consistent with 0 degree of longitude. Then, the latitude of the globe system indicates elevation angle of the glenohumeral joint. The longitude of globe system indicates horizontal adduction or abduction angle of the glenohumeral joint. Arrows indicated direction of motion of the glenohumeral joint as external rotation. ER: External Rotaion of the glenohumeral joint.

To measure strain on the coracohumeral ligament, measurement positions were designated by a combination of positions that have been previously reported in literature and those obtained from our preliminary experiments. Based on the anatomic position of the coracohumeral ligament, which located on the antero-superior aspect of the glenohumeral joint, passive stretching by external rotation was applied to the specimens in each of the designated positions. Range of passive external rotation of the specimens used in this study was from -10° to maximal rotation. In each shoulder position, passive external rotation in 10° increments was applied (Figure 3: Globe-graph of clinical positioning).

In this study, we defined each joint movement using the Globe system.[19-21,31] The scapula plane was set at a longitude of 0 degrees in the Globe system. The ventral and dorsal aspects of the scapula were defined as positive and negative values, respectively, as shown in Figure 3. Further, 0 degrees elevation of the humerus was set at 0 degrees of latitude, with maximum elevation at 180 degrees of latitude. The scapula is protracted 30 degrees in frontal plane toward the rib cage in vivo. In this study, we simulated movement of the glenohumeral joint by fixing the ventral aspect of the scapula with a jig. Flexion and abduction and extension were simulated by 60, -30, and -90 degrees of longitude in the Globe system.

To maintain the tensile properties of the coracohumeral ligament and range of motion of the glenohumeral joint, passive range of motion of the glenohumeral joint was applied 10 times at the end range in each stretching position before the experiment.[32]

Strain on the ligament at each shoulder positions was measured until passive motion reached the terminal range of the glenohumeral joint, which was determined by grade III mobilization after Kaltenborn's procedure.[33] In that grading system, grade III mobilization comprises the manual application of force at a point at which the therapist perceived end-feel of the joint and observed no further stretching of the ligament.

##### Baseline Position

Baseline position: The baseline position of the glenohumeral joint was determined as 0° of elevation with 30° of external rotation on the scapular plane.[10]

In this study, disarticulated scapula from the thorax was fixed on the jig in the frontal plane. Therefore, elevation was designated as glenohumeral abduction in the scapular plane, abduction was designated as glenohumeral abduction with 30 degrees horizontal abduction, and flexion was designated as glenohumeral abduction with 60 degrees horizontal adduction.

##### External Rotation with elevation

The glenohumeral joint was elevated to 0°, 30° or 60° on the scapula plane [11,15-17]. In each shoulder position, passive external rotation was applied from -10° to maximal rotation in 10° increments (Figure 3-A).

##### External Rotation with flexion

The glenohumeral joint was elevated to 30° or 60° on the scapular plane with an additional 60° of horizontal adduction.[17] In each shoulder position, passive external rotation was applied from -10° to maximal rotation in 10° increments (Figure 3-B).

##### External Rotation with abduction

The glenohumeral joint was elevated to 30° or 60° on the scapula plane with an additional 30° of horizontal abduction.[11,13] In each shoulder position, passive external rotation was applied from -10° to maximal rotation in 10° increments (Figure 3-C).

##### External Rotation with extension

The glenohumeral joint was elevated to 30° on the scapula plane with an additional 90° of horizontal abduction.[14] At this shoulder position, passive external rotation was applied from -10° to 50° in maximal rotation in 10° increments (Figure 3-D).

##### External Rotation and adduction with extension

The glenohumeral joint was elevated to 30° on the scapula plane with an additional 90° of horizontal abduction and maximum adduction.[14] At this shoulder position, passive external rotation was applied from -10° to maximal rotation in 10° increments (Figure 3-E).

##### Differences between *in vivo *and *cadavers*

The glenohumeral angle differs in the angle of elevation relative to the thorax. In vivo, the scapula does not rotate from 0 to 30 degrees of elevation of the upper extremity, and after the 30 degrees elevation of the upper extremity, the ratio is 1:1. The scapula is then laterally rotated 30 degrees when the arm is elevated at 90 degrees. For this reason, 60 degrees of elevation in this experiment corresponds to 90 degrees arm elevation in vivo. Therefore, rotation, adduction and abduction at 60 degrees of elevation in this experiment correspond to rotation at 90 degrees of elevation and horizontal adduction and abduction in vivo, respectively.[12,28]

### Identification of reference length (L0) and data analysis

**Figure 4:**
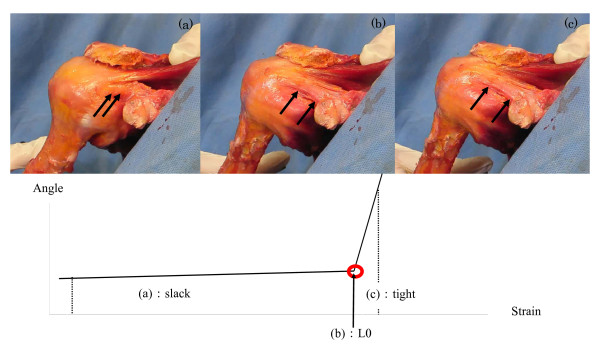
**Identification of the reference length (L0) for the coracohumeral ligament**. Reference length was the length at which the angle-strain curve of the ligament started to indicate a sudden decrease in strain. Photographs (a), (b), (c) indicate the coracohumeral ligament during external rotation at 0° elevation in the scapula plane of the glenohumeral joint; (a) the slack ligament, (b) the ligament at L0, and (c) tight ligament. This arrow is point of change in strain ratio from slack to tight in Figure 4. True strain of the joint capsule can be obtained from this point.

Based on the method in previous reports using cadaver shoulders, reference length (L0) was determined for the ligament.[19,24,34] Reference length was the length at which the angle-strain curve of the ligament started to indicate a sudden decrease in strain. The displacement of the ligament was then defined as the change in length from that at L0. On the basis of data obtained from preliminary experiments, the L0 was determined the designated ligament positions to eliminate slack in the ligament. To measure strain on the ligament and capsule, it is possible that true strain on these tissues can be obtained by subtraction of the slack in the tissues.(Figure 4).

As the stretching procedure described in *Muscle Stretching in Manual Therapy *[35] should be applied to the joint for 10 to 12 seconds after passive motion of the glenohumeral joint has reached the end of the range of movement, each position in our study was maintained for more than 10 seconds until no increase or decrease in strain value was observed.[19,34] The measurements were performed 3 times during each stretching procedure, and a representative value was calculated by averaging the values obtained for each stretching procedure.

### Statistical Analysis

Statistical analysis was performed using SPSS for Windows ver. 11.5 J (SPSS Japan Inc., Tokyo, Japan). Measurement values were analyzed by one-way repeated ANOVA using the raw measurement values for each stretching position. Therefore, reference length 0 was confirmed using the raw measurement values of the distance between the needles at the point where an acute change in the strain was determined. A step-by-step increase in external rotation of the glenohumeral joint was then applied to measure ligament strain. Dunnett's post hoc test was used for multiple comparisons to the raw reference length. The alpha level was set at 0.05.

Positive strain on each ligament was calculated using the following formula:

Strain(%)= ΔL(mm)/L(mm)× 100

Where L is the length between the points at L0, and ΔL is displacement from L0. Strain values greater than 0% indicated positive stretching of the ligament from L0. Values less than 0% indicated no stretching, and are shown as 0% strain.

## Results

The mean terminal range of passive external rotation at each glenohumeral angle as measured by the 3Space Fastrak system was as follows: 53.8 ± 5.3°, 56.1 ± 10.4°, 64.6 ± 22.7° for 0°, 30°, 60°of elevation on the scapula plane, respectively; 58.4 ± 16.0°, 59.9 ± 15.0° for 30°, 60° of flexion, respectively; 55.2 ± 13.4°, 57.4 ± 9.8° for 30°, 60° of abduction, respectively; 51.2 ± 6.8° for 30° of extension; 52.1 ± 13.4° for 30° of extension with adduction. The interclass coefficient of the external rotation angles measured by the manual application of maximal external rotation at each measurement positions is 0.877.

**Figure 5:**
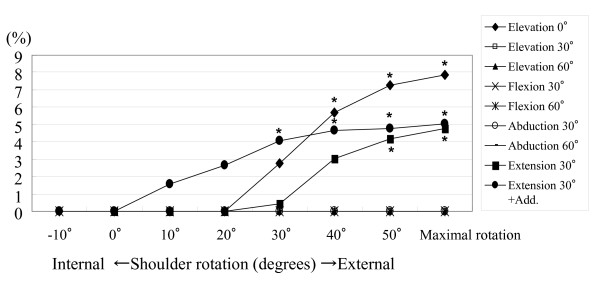
**Patterns of increase of strain on the coracohumeral ligament during passive external rotation in various stretching positions**. The most positive strain on the ligament was obtained from external rotation of greater than 30° in each shoulder position. A 0% strain indicated minus strain values compared to the reference length (L0). Asterisks demonstrate a *P *value of less than 0.05.

The patterns of increase in strain on the coracohumeral ligament during passive external rotation in various stretching positions are shown in Figure 5. Positive strain on the ligament was obtained from external rotation of greater than 30° in each shoulder position.

The positive strain on the coracohumeral ligament obtained at each shoulder position is shown in Table 1. Statistically significant positive strains were obtained at 40°, 50° and maximal external rotation with the arm at 0° elevation, at 50° and maximal external rotation with the arm at 30° extension, and at 30°, 40°, 50° and maximal external rotation with arm at 30° extension with adduction (*P *< 0.05). No positive strain on the coracohumeral ligament was observed by the previously reported stretching positions; i.e., at 90° abduction with external rotation or during flexion with external rotation.

**Table 1:** Strain of the Coracohumeral Ligament and Positions of the Glenohumeral Joint

Shoulder Positions Measured	Reported Authors	In Vitro Measurement	In Vivo Effective Positioning
Elevation 0° with ER	Burkart	Fig. 3-A	Fig. 7-A, D
Elevation 30° with ER		Fig. 3-A	
Elevation 60° with ER	Kuhn	Fig. 3-A	
Flexion 30° with ER	Edelson and Ferrari	Fig. 3-B	
Flexion 60° with ER	Burkart	Fig. 3-B	
Abduction 30° with ER	Edelson and Ferrari	Fig. 3-C	
Abduction 60° with ER		Fig. 3-C	
Extension 30° with ER	Terry and Kelly	Fig. 3-D	Fig. 7-B, E
Extension 30° + Add with ER	Kelly	Fig. 3-E	Fig. 7-C, F

Mean of strain % (standard deviation). 0% strain indicated minus strain values compared to reference length (L0). Ext, external; Rot, rotation.

*: Statistically significant increase in strain compared to L0; P < .05.

**Figure 6:**
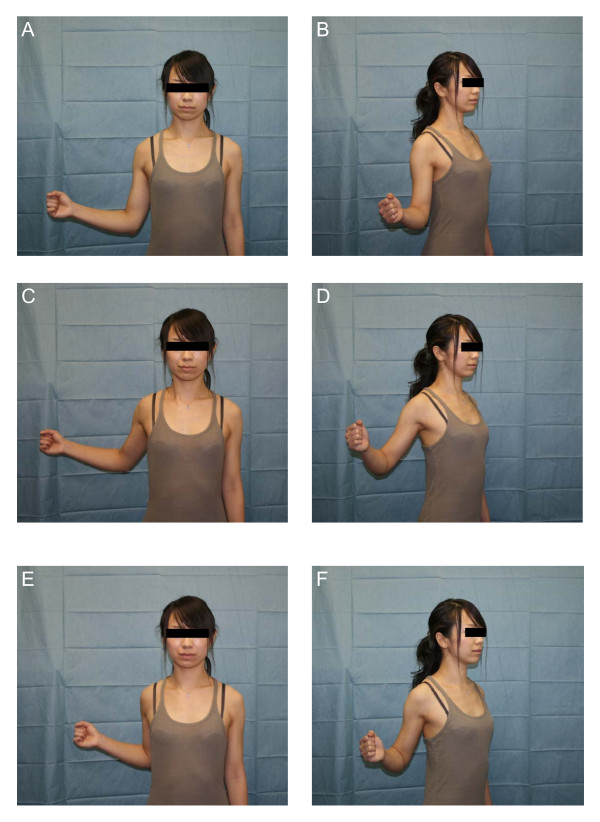
**Potential in vivo stretching positions for the coracohumeral ligament that correspond to positions with significantly increased strain values observed in vitro**. A and B, 0 degrees of elevation with maximum external rotation of the glenohumeral joint. C and D, 30 degrees of extension with maximum external rotation of the glenohumeral joint. E and F, 30 degrees of extension with adduction with maximum external rotation of the glenohumeral joint.

Figure 6 shown potential in vivo stretching positions for the coracohumeral ligament that correspond to the positions demonstrating significantly increased strain values in vitro.

## Discussion

The coracohumeral ligament is a fibrous structure that contributes to the stability of the glenohumeral joint by combined action with the rotator cuff muscles, superior glenohumeral ligament, and capsule. In particular, the coracohumeral ligament functions to prevent inferior subluxation of the humeral head,[13,36] thereby playing an important role in the stabilization of the glenohumeral joint in an upright position. In addition, together with the supraspinatus muscle, subscapularis muscle, superior portion of the glenohumeral ligament, and antero-superior portion of the capsule of the glenohumeral joint, the coracohumeral ligament constitutes the rotator interval. Contracture of the rotator interval has been proved to cause contracture of the glenohumeral joint.[4,26,37]

Burkart et al[11] reported that the coracohumeral ligament originates from the base of the coracoid process and inserts into both the greater tubercle and lesser tubercle of the humerus. Pouliart et al[25] and Tetro et al[26] reported that the coracohumeral ligament was composed of both superficial and deep fibers, and that the superficial fibers insert into the greater tubercle of the humerus and deep fibers insert into the lesser tubercle.[25,26] Moreover, the fibers inserting into the greater tubercle of the humerus comprise the major fibers of the coracohumeral ligament.[11] In this study, we measured the strain on the superficial fibers of the coracohumeral ligament, which insert into the greater tubercle, using a strain gauge.

According to a previous report on the measurement of the tensile properties of the coracohumeral ligament[38], the cross sectional area of the ligament is five times and maximum load is twice to three times that of the superior fibers of the glenohumeral ligament. Gagey et al[8], Neer et al[5] and Ozaki et al[7] have reported that patients with refractory shoulder contracture were successfully treated by surgical resection of the coracohumeral ligament. Therefore, the clarification of effective stretching positions for the coracohumeral ligament is important to treat and prevent shoulder joint contracture by physical treatment.

Various stretching procedures have been reported for the coracohumeral ligament. Burkart et al[11] reported that the coracohumeral ligament restricted the range of motion of external rotation of the glenohumeral joint at 0 degrees of elevation and 50 degrees of abduction. Edelson et al[17] reported that the coracohumeral ligament was tight during flexion and external rotation of the glenohumeral joint, and Ferrari[13] reported that it was tight during external rotation below 60 degrees of abduction. In addition to their studies, Kuhn et al[15] reported that increase in the range of motion of external rotation at 60 degrees of elevation of the glenohumeral joint was recognized when the coracohumeral ligament was released. Terry et al[16] directly measured strain on the coracohumeral ligament in relation to the range of motion of the glenohumeral joint; however, they did not take slack in the ligament into consideration. In their study, flexion and extension of the shoulder joint or external rotation with 0 degrees of elevation were regarded as effective stretching positions for the coracohumeral ligament. Kelley et al[14] reported that positive strain on the coracohumeral ligament was obtained in external rotation with extension, and external rotation with extension plus adduction of the shoulder joint.

In this experiment, a reference length was adopted to measure strain on the coracohumeral ligament. In consequence, positive strain on the coracohumeral ligament was obtained in 9 glenohumeral joint positions; i.e., 40°, 50° and maximum external rotation at 0° elevation, 50° and maximum external rotation at 30° extension, and 30°, 40°, 50° and maximum external rotation at 30° extension with adduction. These results were consistent with the positions (external rotation at 0° elevation) reported by Burkart et al[11], Terry et al[16] and Edelson et al[17]. (Table 2) Moreover, positive strain on the coracohumeral ligament obtained during external rotation with extension, and external rotation with extension and adduction of the shoulder joint was reported by Kelley et al[14]. (Table 2)

**Table 2:** In Vitro Positions of the Glenohumeral Joint for the Strain Measurement of Coracohumeral Ligament and Significant Corresponding In Vivo Stretching Positions of the Shoulder

		-10° Ext. Rot.	0° Ext. Rot.	10° Ext. Rot.	20° Ext. Rot.	30° Ext. Rot.	40° Ext. Rot.	50° Ext. Rot.	Maximal rotation
Elevation	0°	0	0	0	0	2.75(3.17)	5.68(2.16)*	7.27(1.47)*	7.87(1.18)*
	30°	0	0	0	0	0	0	0	0
	60°	0	0	0	0	0	0	0	0
Flexion	30°	0	0	0	0	0	0	0	0
	60°	0	0	0	0	0	0	0	0
Abduction	30°	0	0	0	0	0	0	0	0
	60°	0	0	0	0	0	0	0	0
Extension	30°	0	0	0	0	0.43(2.51)	3.04(1.34)	4.20(1.65)*	4.79(1.67)*
	30° + Add.	0	0	1.57(3.65)	2.65(3.23)	4.09(3.23)*	4.67(3.40)*	4.78(4.42)*	5.05(5.20)*

ER, external rotation; Add, Adduction.

The strain on the coracohumeral ligament in this study ranged from 4.20% to 7.87%. Although these strain values are compatible with those obtained from the lineal region of a stress-strain curve of a tendon or ligament; in this region micro ruptures in the ligament and tendon occur[39], the maximal strain on the inferior glenohumeral ligament was reported by Bigliani et al[29] to be 10.9%. Therefore, stretching of the coracohumeral ligament within the range of 4.20% to 7.87% is considered to present little risk of injury to the ligament. Stretching procedures obtained from this mechanical study have the potential to stretch the coracohumeral ligament with safety and are thought to be useful for the treatment of shoulder contracture.

There are several limitations to this study. First, because the specimens were harvested from aged cadavers, the range of motion and mechanical properties of the specimens might be different from those of specimens from younger adults, with the strain on the ligament observed in the aged cadavers likely to be smaller than that on ligament in younger adults.[40]

Second, the interclass coefficient of the measured external rotation angles by the manually application of maximal external rotation at each measurement position was 0.877 in this study, which is good for criterion of interclass coefficient confidence. However, it is true that the rotational torque of the shoulder produced by the manual application using grade III mobilization after Kaltenborn's procedure may fluctuate. We will establish a quantitative measurement system for the applied rotational torque of the shoulder joint using a torque gauge in future studies.

Third, in this study, though humeral head subluxation was carefully observed by three researchers and no visible and palpable subluxation was detected during experiment, minor translations of the humeral head might have occurred without being noticed.

## Conclusions

Stretching positions of the coracohumeral ligament in which significantly larger strains were obtained were 0° elevation with 40°, 50° and maximum passive external rotation, 30° extension with 50° and maximum external rotation, and 30° extension + adduction with 30°, 40°, 50° and maximum external rotation. No positive strain was observed on the coracohumeral ligament using previously reported stretching positions; i.e., 90° abduction with external rotation or flexion with external rotation. Stretching procedures obtained from this in vitro mechanical study have the potential to stretch the coracohumeral ligament with considerable safety in vivo and are thought to be useful for the treatment of shoulder contracture.

## Competing interests

The authors declare that they have no competing interests.

## Authors' contributions

T I and M A provided concept/idea/research design. T I provided writing and data analysis. T I and Y T provided data collection. E U provided project management. S M provided facilities/equipment and consultation. M F and D S managed of fresh/frozen cadaver specimens. All authors read and approved the final manuscript.
